# *Aspergillus* population diversity and its role in aflatoxin contamination of cashew nuts from coastal Kenya

**DOI:** 10.1371/journal.pone.0292519

**Published:** 2024-01-25

**Authors:** Colletah Rhoda Musangi, Bicko Steve Juma, Dennis Wamalabe Mukhebi, Everlyne Moraa Isoe, Cromwell Mwiti Kibiti, Wilton Mwema Mbinda

**Affiliations:** 1 Department of Biochemistry and Biotechnology, Pwani University, Kilifi, Kenya; 2 Pwani University Bioscience Research Centre (PUBReC), Pwani University, Kilifi, Kenya; 3 Department of Pure and Applied Sciences, Technical University of Mombasa, Mombasa, Kenya; Tocklai Tea Research Institute, INDIA

## Abstract

Cashew nuts are among the main cash crops in coastal Kenya, due in large part to their high nutritional value. Unfortunately, they also make them highly susceptible to mold contamination, resulting in biodeterioration of the nutritional value and potential contamination with toxic secondary metabolites, such as aflatoxins, that cause them to be rejected for sale at the market. We determined the population diversity of the *Aspergillu*s species and their role in aflatoxin contamination in cashew nuts in selected coastal regions of Kenya. Fifty raw cashew nut samples were collected from post-harvest storage facilities across three counties in Kenya’s coastal region and examined for moisture content and the presence of *Aspergillus* fungi. About 63 presumptive isolates were recovered from the cashew nuts. ITS and 28S rDNA regions were sequenced. The *aflD*, *aflM* and *aflR* genes were amplified to identify the potentially aflatoxigenic from the Aspergillus isolates. The Aflatoxins’ presence on the isolates was screened using UV and the ammonia vapour test on coconut milk agar and validated using ELISA assay. A comparison of cashew moisture content between the three counties sampled revealed a significant difference. Sixty-three isolates were recovered and identified to section based on morphological characters and their respective ITS regions were used to obtain species identifications. Three sections from the genus were represented, *Flavi and Nigri*, and *Terrei* with isolates from the section *Nigri* having slightly greater abundance (n = 35). The *aflD*, *aflM* and *aflR* genes were amplified for all isolates to assess the presence of the aflatoxin biosynthesis pathway, indicating the potential for aflatoxin production. Less than half of the *Aspergillus* isolates (39.68%) contained the aflatoxin pathway genes, while 22.22% isolates were aflatoxigenic, which included only the section *Flavi* isolates. Section *Flav*i isolates identification was confirmed by calmodulin gene. The presence of species from *Aspergillus* section *Flavi* and section *Nigri* indicate the potential for aflatoxin or ochratoxin in the cashew nuts. The study established a foundation for future investigations of the fungi and mycotoxins contaminating cashew nuts in Kenya, which necessitates developing strategies to prevent infection by mycotoxigenic fungi, especially during the storage and processing phases.

## Introduction

Cashew trees (*Anacardium occidentale*) are among the main cash crops in Coastal Kenya. They are grown by small-holder farmers in Kilifi, Kwale, Mombasa, Tana River and Lamu counties for their nuts [1]. Processed cashew nuts and cashew nut products are among the most widely traded nut goods globally and their demand is rising rapidly [2]. Despite, the increasing global demand, cashew nut production in Kenya has suffered numerous constraints which are linked to the agronomic, processing, and marketing, thereby affecting the quality and quantity of the harvested nuts [2]. Due to high post-harvest prevalence of pests and other diseases, yield losses have been increasingly experienced. Additionally, a dramatic decline in cashew nut marketability has occurred due to mycotoxin contamination which renders them unsafe for consumption and sale [3].

Cashew nuts are vulnerable to fungal infestation due to high levels of protein, minerals, and fats [4]. Infection commonly occurs due to poor handling and storage of the harvested nuts [5] although it can happen at other stages such as pre-harvest (while still on the trees) or during harvest [6]. When the seeds are not properly dried before storage to moisture content of < 8% is considered safe for raw cashew nuts, they facilitate fungal growth and infestation [3]. Additionally, storage conditions involving high humidity and temperatures encourage fungal growth in cashew nuts and possible mycotoxin contamination [7]. The mycotoxigenic fungi may produce a multitude of mycotoxins such as aflatoxins, ochratoxin A, deoxynivalenol, zearalenone, fumonisins, patulin, and trichothecene T-2 and HT-2 toxins [8]. Of these, aflatoxins (AFs) are the most important due to their carcinogenic and genotoxic effects [9]. Aflatoxins are produced by species within *Aspergillus* section *Flavi*, including *A*. *flavus*, *A*. *parasiticus*, *A*. *nomius* and *Aspergillus* species that produce the small sclerotia. These fungi are often implicated as causal agents of aflatoxin contamination of crops and food in Africa, and globally [10]. Taking proper precautions during collection and post-harvest handling of cashew nuts could substantially reduce the risk of infestation by mycotoxigenic fungi [11]. Aflatoxigenic fungi can grow well even at warm temperatures between 35 to 40°C; however, the optimal production of aflatoxins has been recorded between 28 and 31°C [12, 13]. Additionally, humidity above 85% favors their growth especially in storage facilities, encouraging aflatoxin production [14]. Aflatoxin contamination of cashew nuts and other food crops is major problem in Africa, especially in the coastal agro-ecological regions, because the climatic conditions are suitable for the growth of fungi and the synthesis of the toxins [15]. In the tropical and subtropical areas of the world, aflatoxin contamination has been reported in milk, spices, maize, rice, peanuts, tree nuts including almonds, Brazil nuts and cashew nuts [16–18].

*Aspergilli* are filamentous fungi that thrive as saprophytes and are found on variety of substrates and environments [19]. The genus is subdivided into 22 sections, half of which are clinically relevant species [20]. *Aspergillus* section *Flavi* is comprised of both aflatoxigenic and non-aflatoxigenic species [21]. Although the numbers of recognized aflatoxigenic species is expanding, *A*. *flavus*, *A*. *parasiticus* and *A*. *nomius* are the most widely recognized aflatoxin producers [22, 23]. Moreover, in section *Flavi*, *A*. *arachdicola*, *A*. *pseudotamarii*, *A*. *bombycis*, *A*. *toxicarius (A*. *parasiticus)*, *A*. *aflatoxiformans* (formerly *A*. *parvisclerotigenus*) and *A*. *minisclerotigenes* have been documented as aflatoxin producers [24, 25]. Their morphological and molecular characterizations have been conducted in association with maize [26], rice [27], cassava, millet and sorghum [28], ground nuts and tree nuts [29, 30], peanuts [25] and wheat [31]. Previous findings involving their presence in cashew nuts have also been reported by [32] and [6] although the quantification levels were ascertained in both studies. However, no studies have been conducted to characterize the aflatoxin-producing fungi in cashew nuts from the Kenyan. Aflatoxin contamination in maize is endemic in Kenya. For instance, in April 2004, one of the largest aflatoxicosis outbreaks occurred, resulting in 317 cases and 125 deaths [33]. Aflatoxin-producers are not to be host specific and species detected in maize could infect cashews nuts with high levels of aflatoxins [34]. In this study, we report that cashew nuts from coastal Kenya were infested by *Aspergillus* species that to produce aflatoxins and may have the potential to secrete of other mycotoxins, necessitating vigilance and continued research in mycotoxigenic fungi for guaranteeing consumer health and enhancing livelihoods and economics related to cashew nuts.

## Materials and methods

### Sample collection

Cashew nut samples were collected from storage containers in the Kilifi, Kwale and Lamu counties along the coastal region of Kenya using a stratified random sampling method as described by [35]. The cashew nut producers were divided into small areas (strata) according to their geographic locations in each county. From each location, at least three samples were collected and thoroughly mixed for a combined weight of 1 kg. A total of 19, 21, and 10 samples were collected from Kwale, Kilifi, and Lamu counties, respectively. The kernels were then wrapped in labelled Ziplock bags, sealed and transported to Pwani University Biosciences Research Centre (PUBReC), Pwani University, Kilifi, Kenya for downstream analysis. Upon arrival, each sample was divided into two equal halves; one half was stored at 4°C and the other half was stored at room temperature.

### Moisture content evaluation

Moisture content was determined using the oven drying method as described by [36] with some modifications. Cashew nut samples with shell were measured for 100 g from each sample and placed in a glass Petri dish of known weight (W0) and the initial weight (W1) of each sample was determined. The Petri dishes were then heated to 105°C in the oven for drying overnight. Drying of the cashew nuts continued with hourly weight measurements until the reading of each sample was constant indicating all the moisture had been completely removed. The dry weight (W2) was used to calculate the moisture content according to the formula:

$$Moisture\;content\;(\%)=\frac{W1-W2}{W1-W0}\times 100$$

The percentage of moisture content was calculated for all 50 samples, which were then grouped according to their sampling areas to calculate the means of each county separately.

### Fungal isolation

Cashew nuts were direct plated to recover isolates of *Aspergillus* species as described by [37]. Whole cashew nuts were surface sterilized by immersion in 70% ethanol followed by three rinses with sterile distilled water. The excess sterile distilled water was removed by squeezing the kernels between sterile filter paper. Sterilized cashew shells were then cut in four directions using a sterile scalpel to help remove the outer shell and expose the cashew kernel within. The kernels were then cut into several pieces of approximately the same size (3 mm x 4 mm) using a sterile scalpel. The particles were directly placed on modified Rose Bengal Agar (MRBA) medium [38] in 60 mm × 15 mm Petri dishes, with three pieces per plate, and a technical replication and incubated at 30°C for 7 days in darkness. The number of infected particles were counted and expressed as a percentage of the total number of kernel pieces that were plated. Fungi growing on the kernels were transferred to Water Agar (WA) medium and incubated for 3 days at 27°C in the light for mono-conidiation. The resulting hyphae were viewed in the light, and further cultured on Potato Dextrose Agar (PDA) and Malt Extract Agar media (MEA) at 25°C for 7 days in the light to isolate pure cultures. The percentage growth rate of the aspergillus isolates growth were subjected to anomality test using qqplot of normality test in R package. The percentage growth rate of the *Aspergilli* isolates was then compared to the moisture content to determine whether their correlation is positive or negative.

### Morphological characterization of *Aspergillus* isolates

Examination of macro-morphological characters was based on the mycelium growth pattern, the presence of conidia, sclerotia and hyphae pigmentation. All the macroscopic and microscopic features were compared to the synoptic keys of identification of the fungi isolated as described by [39]. Colony color allowed for rapid grouping of isolates based on section, whereby the brown and black colonies belonged to *Aspergillus* section *Nigri* and the greenish colonies belonged to section *Flavi*. To examine micro-morphological characters, vegetative tissue from each 7-day old culture was mounted on a glass slide and stained with lactophenol cotton blue for micro-morphological examination using light microscopy at 40X and 100X magnifications. Slides were examined on a Zeiss Primo Star dissecting light microscope mounted with the ZEISS Axiocam ERc 5s Microscope Camera 5mp. Slides exhibiting good micro-morphological characters were made into permanent slides for future reference. The isolates were characterized based on size, shape and ornamentation of microscopic structures such as conidiophore stipes, vesicles and conidia.

The colonies were grouped according to their distinct colors and the members of the different sections, species and strains were confirmed using the microscopic features. These included the seriation; either biseriate, uniseriate or biseriate and uniseriate, conidial head shape; either columnar or globose, and the diameter and shape of the vesicle. Other micro-morphological features used to distinguish the species were the conidiophore shape towards the vesicle, the conidia head size and the arrangement of the phialides into the metulae. The occurrence of each species was then expressed in percentage of the species compared to the total number of isolates using the formula:

$$Occurance\;of\;species\;(\%)=\frac{\text{No}.\;\text{of}\;\text{isolates}\;\text{of}\;\text{a}\;\text{species}}{\text{Total}\;\text{no}.\;\text{of}\;\text{isolates}}\times 100$$

### Molecular confirmation of species

Genomic DNA (gDNA) from 63 pure isolates cultured in PDA medium was extracted from fresh scraped culture using a sterile scalpel blade. The scraped tissues were crushed in liquid nitrogen to fine powder. The gDNA was extracted using Qiagen DNeasy Plant Mini according to the manufacturer’s described protocol. The extracted gDNA was then used for PCR amplification using gene/domain specific primers (Supporting information 1 in S1 File). The internal transcribed spacer region (ITS1/4 primers), as well as the D1/D2 domains of the nuclear (NL) region of the 28S rDNA large subunit (NL1/4 primers) were used to confirm the morphological identifications of our *Aspergillus* isolates [40]. Both regions were also used for diversity estimation studies following the protocol of [40]. The presence of the aflatoxin biosynthesis pathway was determined in all the isolates using primers specific for three pathway genes (*nor-1*/*aflD*, *ver-1*/*aflM* and *aflR*) based on a previous study that suggested these genes could be used to discern between aflatoxigenic and non-aflatoxigenic *Aspergilli* [41]. The PCR products of the ITS and 28S rDNA amplicons for all 63 isolates were sequenced using Sanger sequencing technology. The ab1 files for each region/domain were analyzed separately. The nucleotide sequences were then queried using the BLASTn tool from the NCBI database to identify the fungi related to the sequences. The 21 isolates identified as *Aspergillus* section *Flavi* from the BLASTn analysis of the 63 isolates were further confirmed by sequencing of calmodulin gene (Cmd5/6 primers) [40] (Supporting information 1 in S1 File). The phylogenetic relatedness of the *Aspergillus* isolates was inferred using the maximum likelihood approach. Newick trees were generated using the Fasttree package (https://help.rc.ufl.edu/doc/FastTree). A bootstrap replication of 1000 was used. The trees were annotated using the ggtree package in R package.

### Screening of isolates for aflatoxin presence

Pure isolates obtained from PDA/MEA media were screened for their aflatoxigenicity on coconut milk agar (CMA) medium [42]. Each isolate was inoculated in freshly prepared CMA and incubated at 28°C for 7 days. Further, the isolates were exposed to UV light to identify those that had produced aflatoxins. Isolates that absorbed and emitted very bright, bright, or weak UV fluorescence at 365 nm were considered to be capable of producing aflatoxins [26]. After incubation, the Petri plates were turned upside down and 2 mL of concentrated ammonia solution was poured into the lid of inverted culture plate and kept for 10–15 minutes to release ammonia vapour. On exposure of culture to ammonia vapour the color development was recorded. Any color change from yellow to dark yellow, pink, or reddish brown was considered as an indicator of positive aflatoxin production [43]. Isolates that were aflatoxigenic were validated for total aflatoxins using the enzyme-linked immunosorbent assay using the 96 well Thermo Fisher ELISA kit. All the samples tested positive, and the intensity of the yellow color was measured by microplate reader at 450 nm. The results were calculated as:

$$\frac{B}{B0}(\text{\%})=\frac{\text{standard}\;(\text{or}\;\text{sample})\text{absorbance}}{\text{standard}\;0\;\text{absorbance}}X\;100$$

And the concentrations of individual samples interpolated from the calibration curve generated from the AF-B1 standards.

### Data analysis

The mean moisture content was calculated for all the 13 locations, followed by comparison for differences within the agroecological regions and among the selected counties of the Kenyan coast. For the statistical significance test, a normalcy test was carried out using Shapiro wilk test that showed a normal distribution (p> 0.05) of the data. The moisture content means of Kilifi and Kwale county were analyzed using one-way ANOVA (*p <0*.*05*) followed by HSD test, while Lamu data were analyzed using the two tailed un-paired student t- test at p< 0.05 in R software (v4.2.1). The comparison of the moisture content means among the Kilifi, Lamu and Kwale regions was done using the Kruskall-Wallis test (*p <0*.*05*), followed by a post- hoc test at a *p<0*.*05*. Diversity distance estimations of the *Aspergillus* isolates sequences were determined newick Tree was generated using the Fasttree package (https://help.rc.ufl.edu/doc/FastTree). The tree was annotated using the ggtree package in R [44]. The haplotype diversity estimation within and among the three counties and analysis of molecular variance (AMOVA) were determined using Arlequin version 3.5 [45].

## Results

### Moisture content evaluation

**Fig 1:**
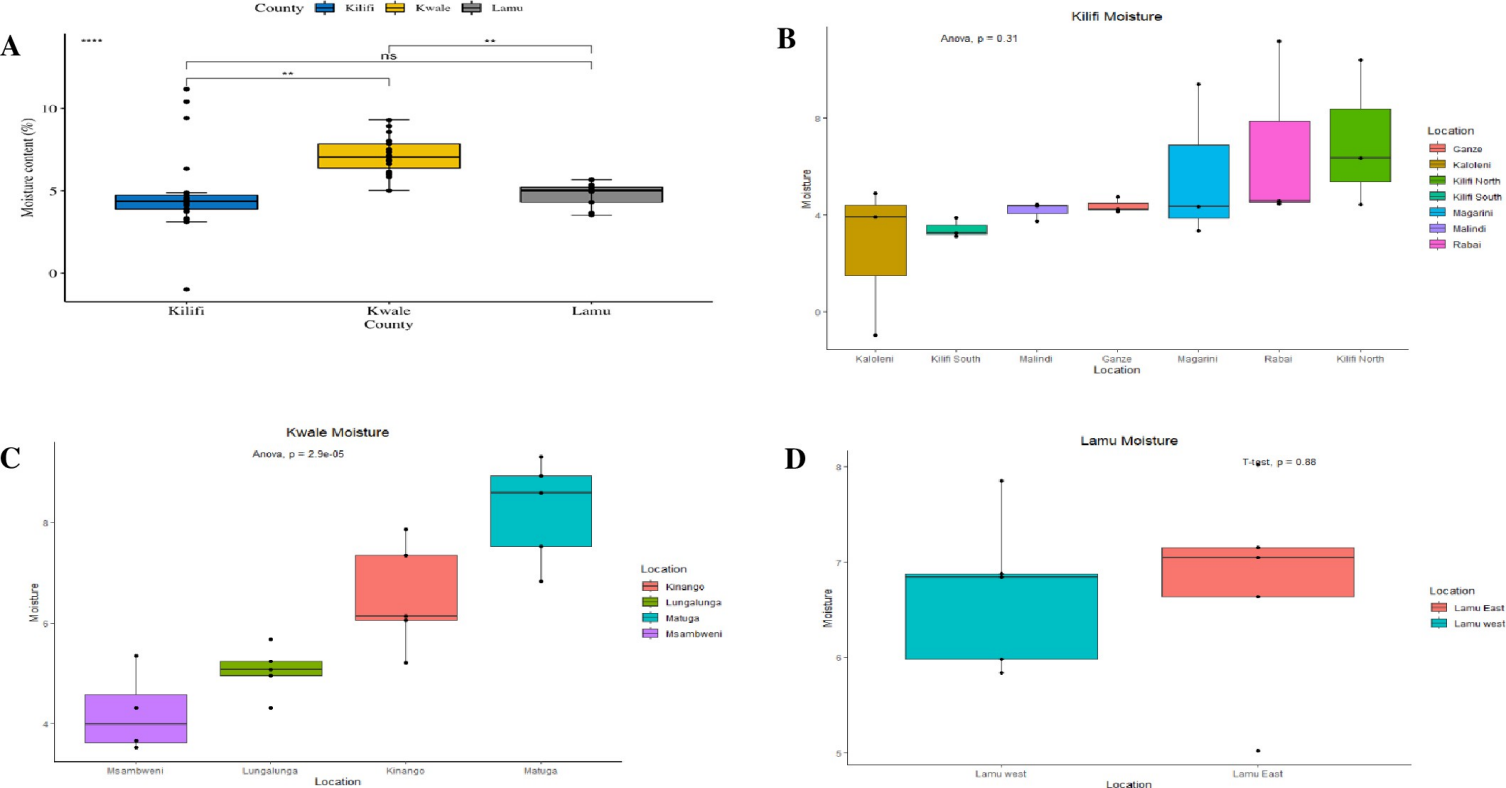
Box plots comparing the average moisture content of cashew nuts between (A) and within (B, C and D) each of the three sampling counties. Between-county values compared using the Kruskal Wallis test, followed by the Post Hoc test (p < 0.05). Within-county values compared using a one-way ANOVA, while Lamu County values were compared sing T-test (p < 0.05).

**Fig 2:**
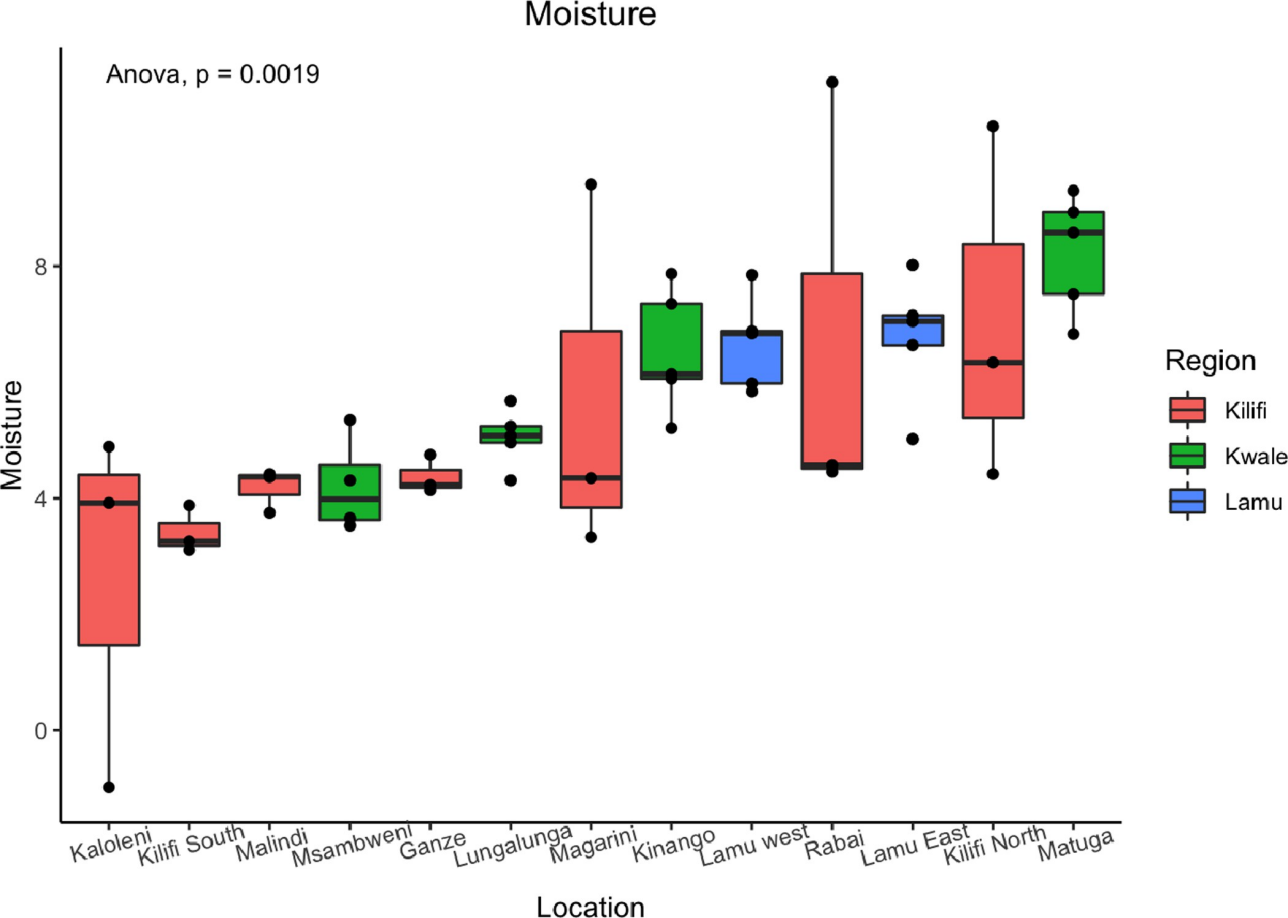
Box plots comparing the average moisture content of cashew nuts from each of the 13 sampling locations. The means were assessed for statistically significant differences by one-way ANOVA (p < 0.05), yielding a test statistic of (F) = 3.423 and p-value of p = 0.002. To confirm the significantly different pairs, the Tukey’s Honest Significant Difference (HSD) test was performed. * p < 0.05, ** p < 0.01, no asterisk indicates lack of significance.

Comparing the three counties, cashew nuts collected from Kwale, Lamu and Kilifi showed significant differences (*p* < 0.05) in moisture content (Fig 1A). Lamu County had the lowest moisture content (4.73 ± 1.50%), while Kwale County had the highest moisture content (7.15 ± 0.25%) (Fig 1A; Supporting information 2 in S1 File). A significant difference in moisture content was found between Kilifi and Kwale (*p* = 0.00113) and between Kwale and Lamu (*p* = 0.00556) (Fig 2). However, there was no significant difference in moisture content of the cashew nuts from Lamu and Kilifi counties (*p* = 0.981) (Fig 1A). The mean moisture for the cashew nuts collected from the three counties was 5.71 ± 0.81% (Supporting information 2 in S1 File). Assessing differences in moisture content within each county (across sampling locations), the moisture content from the seven locations across Kilifi County did not have significant differences (Fig 1B). The lowest moisture content mean was recorded for cashew nuts collected from Kaloleni (2.61 ± 1.74%), while the highest moisture content mean was recorded in cashew nuts collected from Kilifi North (7.06 ± 1.77%) (Fig 2; Supporting information 2 in S1 File). The average moisture content of the cashew nuts from Kilifi County was 4.87 ± 0.99% (Supporting information 2 in S1 File). Comparison of moisture content means across the four sampling locations within Kwale County also revealed no significant differences (Fig 1C) with the least moisture content mean recorded for cashew nuts from Msambweni (6.68 ±0.63%) while the highest mean was recorded in cashew nuts from Matuga (8.096 ±0.53%) (Fig 1C, Supporting information 2 in S1 File). The overall moisture content mean of the cashew nuts from Kwale County was 7.15 ± 0.25% (Supporting information 2 in S1 File). The moisture content means in the cashew nuts collected from the two sampling locations (Lamu East and Lamu West) of Lamu County revealed no significant difference (Fig 1D). Cashew nuts from Lamu East had a lower moisture content mean of 4.41 ±0.38% compared to those from Lamu West which recorded moisture content mean of 5.05 ±0.22% (Supporting information 2 in S1 File). The overall mean of the moisture of the cashew nuts from Lamu County was 4.73 ±1.49% (Supporting information 2 in S1 File). Results of the mean moisture content of the 50 cashew nut samples collected from all 13 locations showed significant differences between some but not all locations (Fig 2). Pair-wise comparison of the mean moisture content from the sampling locations shows no significant difference at (*p<0*.*05*) from the 74 of the 78 location pairings, however, there was significant difference observed in only 4 of the 78 location pairings (*p = 0*.*0019*) as demonstrated in the sampling locations of Matuga (Kwale) and Kaloleni (Kilifi) *p* = 0.00564, and Matuga (Kwale) and Kilifi South (Kilifi) *p* = 0.0317 (Fig 2).

### Morphological characterization of *Aspergillus* isolates

Different *Aspergillus* species were isolated from MRBA selective medium resulting to 63 distinct isolates (Supporting information 3 in S1 File) which were cultured onto each of two media (PDA and MEA) for morphological characterizations. We observed distinct mycelial pigmentations such as white, yellow, black, and green on PDA (Supporting information 4 in S1 File) and on MEA (Supporting information 5 in S1 File). Based on colony colors, it was possible to group the isolates into one of two possible sections, *Flavi* (greens and yellows) and *Nigri* (browns and blacks). Macro-morphological characters allowed us to putatively identify isolates to species which included *A*. *parasiticus*, *A*. *flavus* and *A*. *oryzae* from section *Flavi*, and *A*. *niger* and *A*. *tubingensis* from section *Nigri*. We then examined micro-morphological characters to further improve our species identifications with 100X magnification facilitating better differentiation of conidiophore structures and conidium characters (Supporting information 6 in S1 File).

**Fig 3:**
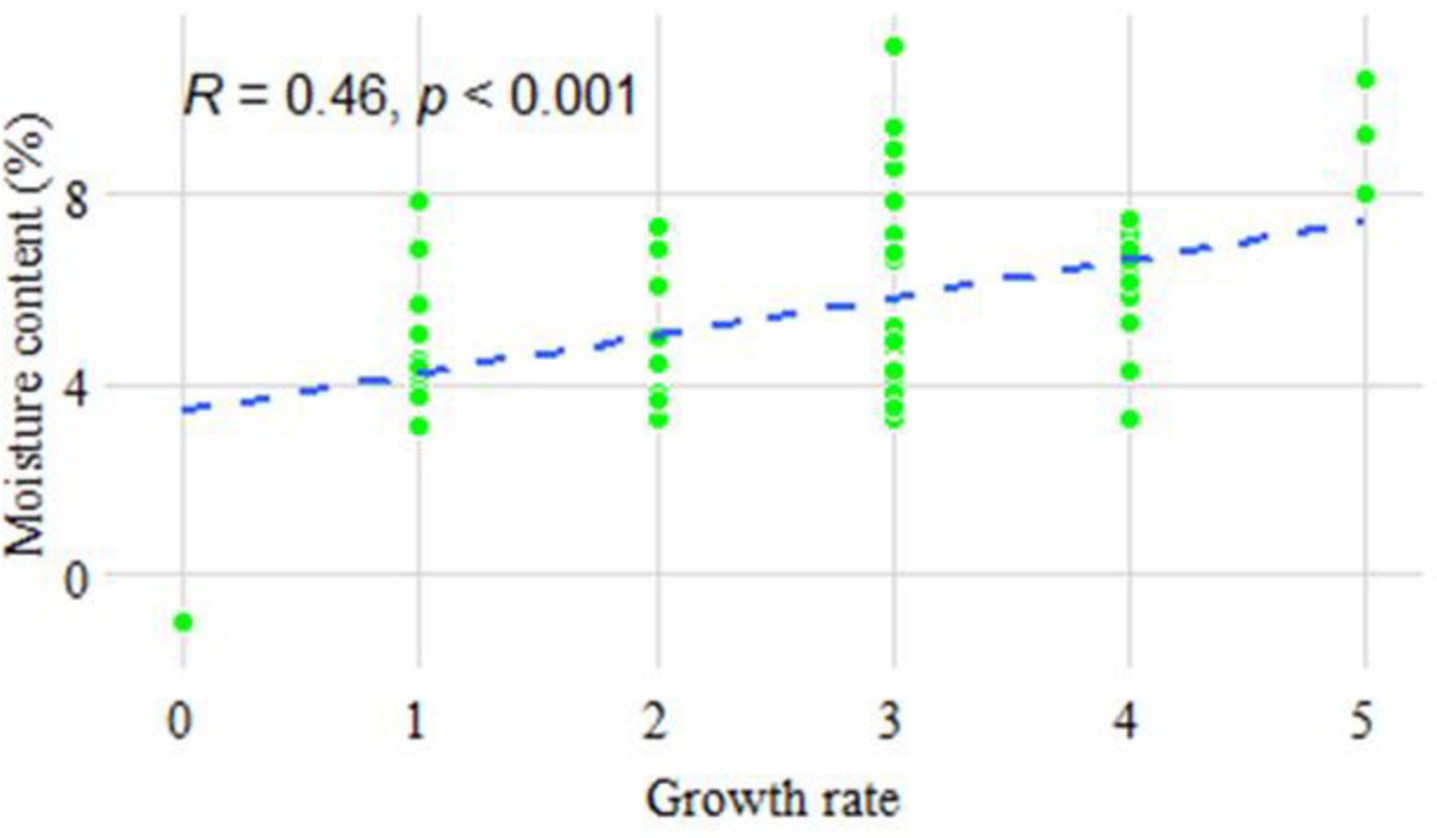
Graph showing the correlation between cashew kernel moisture content with growth rates of *Aspergillus* isolates. Data was evaluated using the Pearson correlation method and resulted in r = 0.4582 at 95% confidence interval, R square = 0.2100, p-value = 0.0001. The correlation was Significant at (alpha = 0.05) for the number of XY Pairs = 65. The growth rates were measured in mm/day.

Occurrence of the species per county showed that *A*. *oryzae* was not recovered from both Kwale and Kilifi counties while only one (100.00%) isolate of *A*. *oryzae* was recovered in Lamu County (Table 1). The highest proportion of all species recovered in each county was *A*. *parasiticus* in both Kilifi (45.45% of the total isolates) and Lamu (36.36% of the total isolates). *A*. *niger* was most prevalent in Kwale with 41.38% of the total isolates (Table 1). The moisture content of the cashew nuts had a positive correlation (R^2^ = 0.21) with the growth of the *Aspergillus* isolates obtained from the PDA media. The cashew nuts with lower moisture content showed slower growth rate of the isolates while the ones with higher moisture content showed faster growth rate of the fungi (Fig 3).

**Table 1:** Occurrence of *Aspergillus* species from each sampling county based on morphological identification.

Population	Kilifi	Kwale	Lamu	Total
Species	No. of isolates (^a^)	No. of isolates (^a^)	No. of isolates (^a^)	No. of isolates (^a^)
*A*. *niger*	11 (37.93%)	12 (41.38%)	6 (20.69%)	29 (46.03%)
*A*. *tubingensis*	3 (50.00%)	2 (33.33%)	1 (16.67%)	6 (9.52%)
*A*. *flavus*	6 (37.50%)	7 (43.75%)	3 (18.75%)	16 (25.39%)
*A*. *parasiticus*	5 (45.45%)	4 (36.36%)	2 (18.10%)	11 (17.46%)
*A*. *oryzae*	0 (0.00%)	0 (0.00%)	1 (100%)	1 (1.59%)
Total	25 (39.68%)	25 (39.68%)	13 (20.63%)	63 (100%)

^a^ Percentages shown in parentheses were calculated based on the total number of species identified out of the total number of isolates.

### Molecular confirmation of species identification

**Fig 4:**
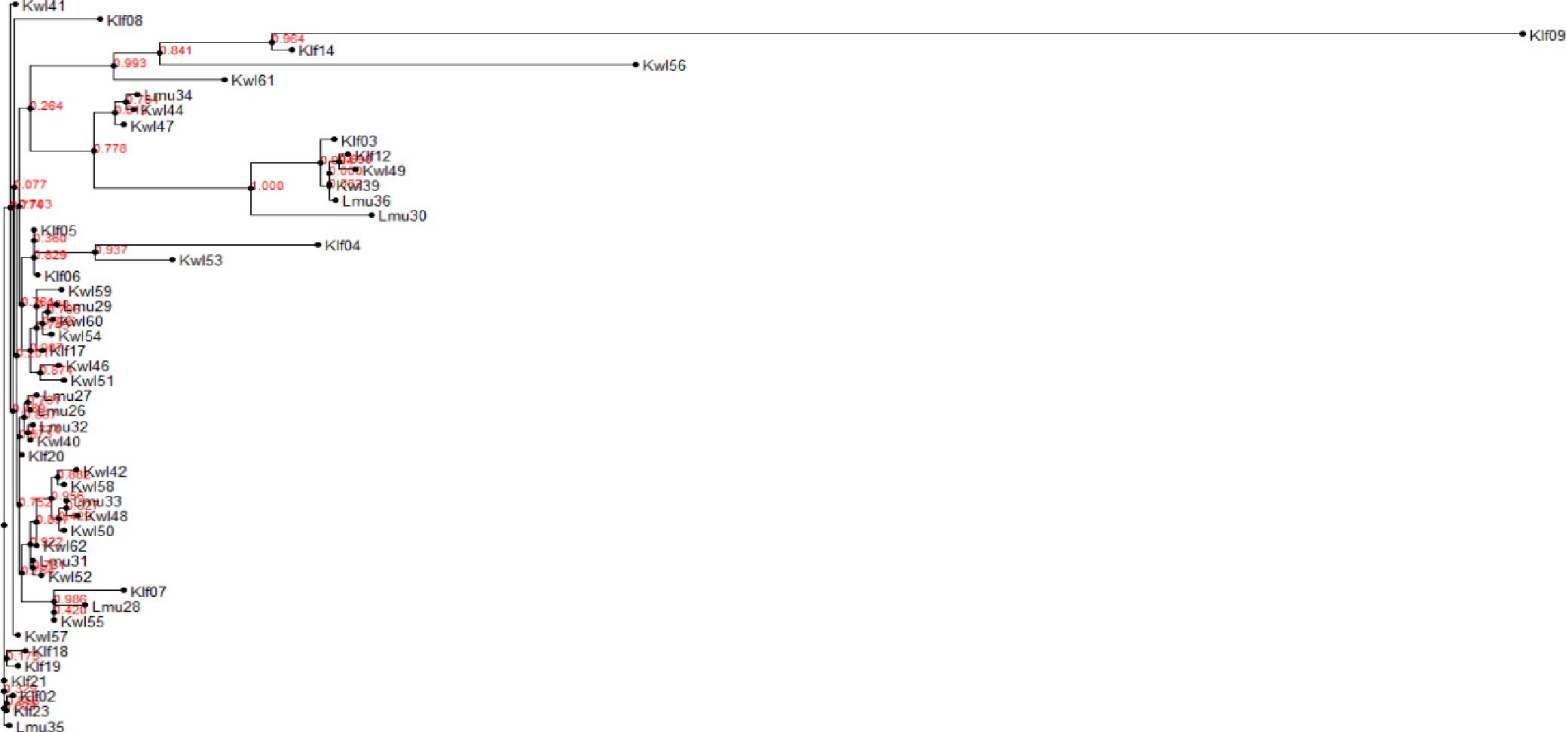
Phylogeny of *Aspergillus* isolates recovered from cashew nuts using nucleotide sequences from A. 28S rDNA region. The phylogeny was inferred using maximum likelihood method of newick tree generated using the Fasttree package. A bootstrap replication of 1000 was used.

**Fig 5:**
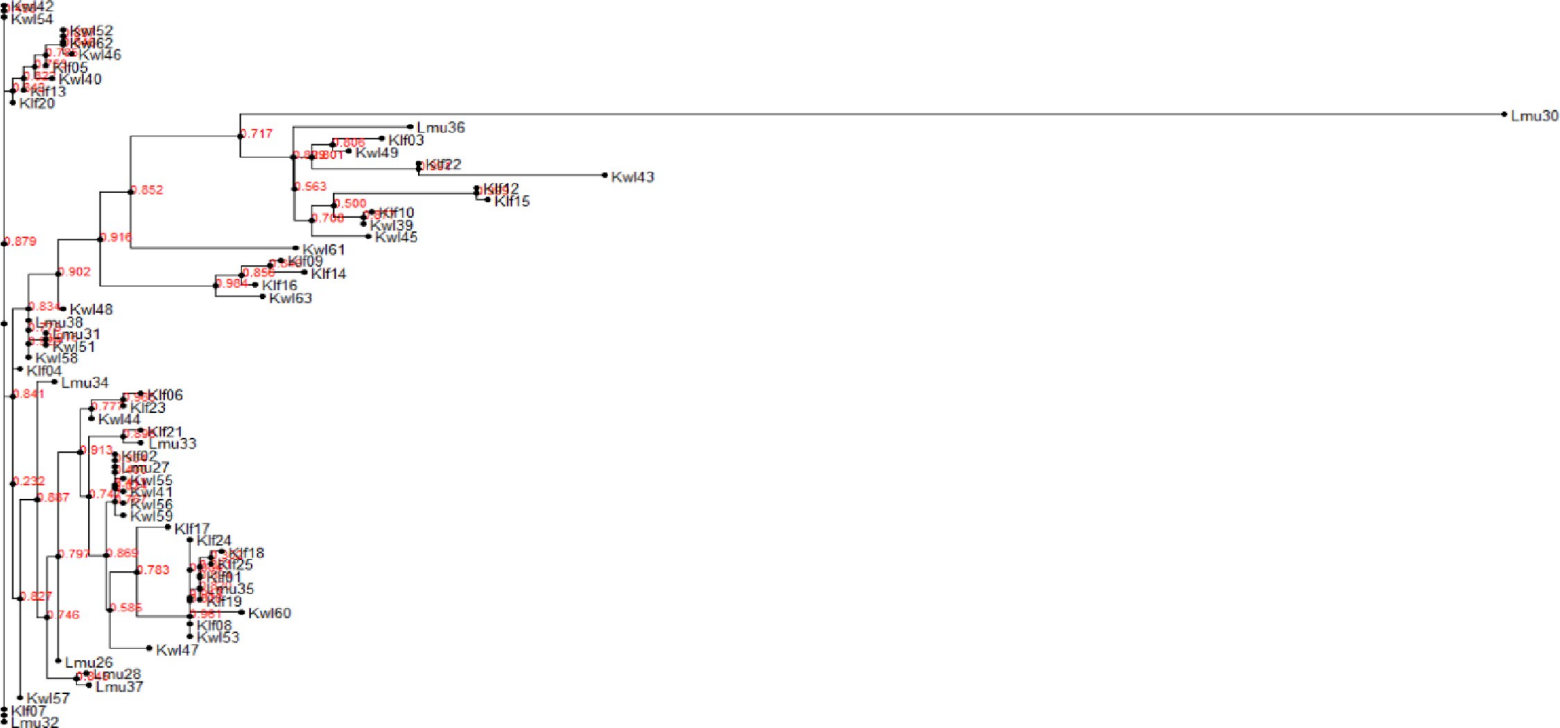
Phylogeny of *Aspergillus* isolates recovered from cashew nuts using nucleotide sequences from ITS region. The phylogeny was inferred using maximum likelihood method of newick tree generated using the Fasttree package. A bootstrap replication of 1000 was used.

**Fig 6:**
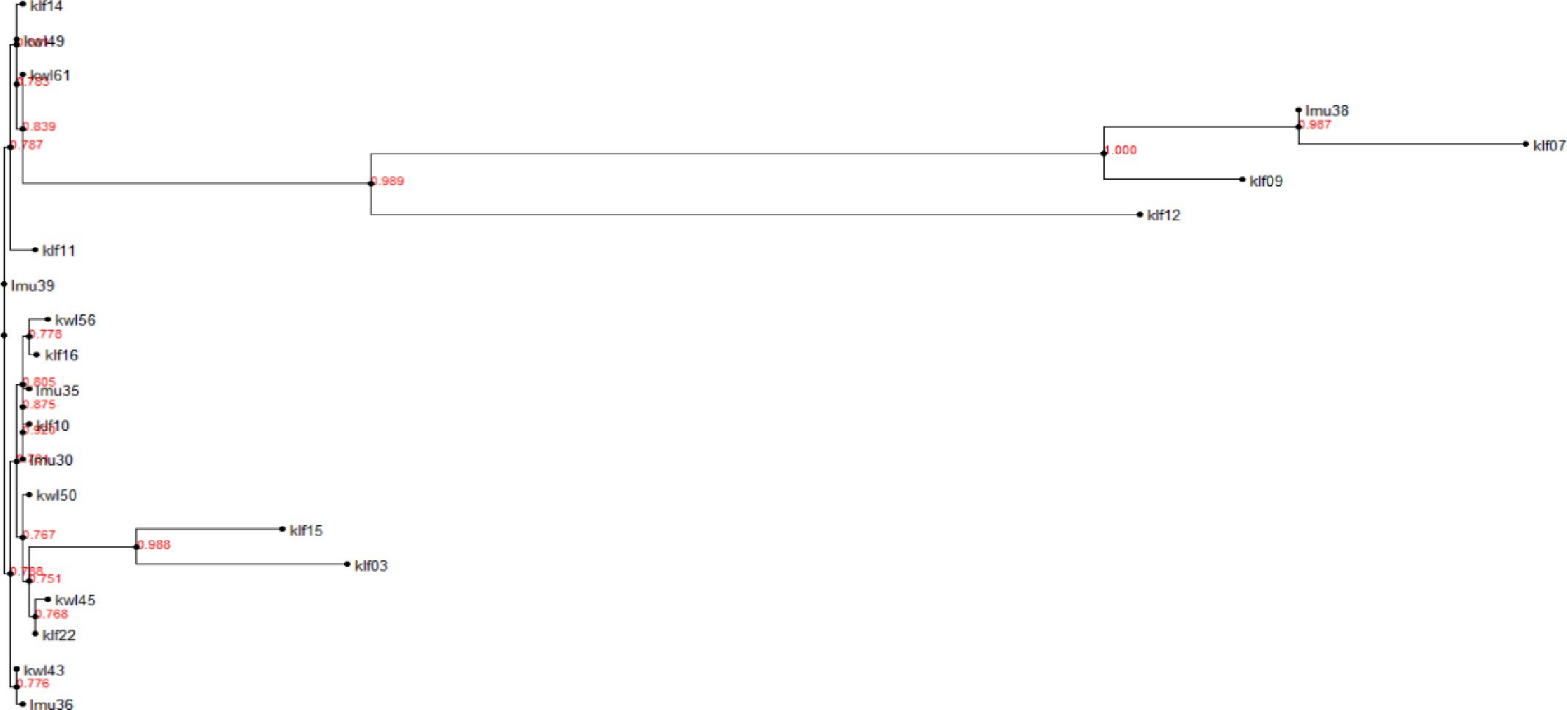
Phylogenetic tree of *Aspergillu*s species section *Flavi* confirmed by use of calmodulin gene. The tree shows *Aspergillu*s isolates previously detected by ITS and 28S rDNA regions. The phylogeny was inferred using maximum likelihood method of newick tree generated using the Fasttree package. A bootstrap replication of 1000 was used.

PCR amplification of the ITS (Supporting information 7 in S1 File) and 28S rDNA regions (Supporting information 8 in S1 File) for our 63 *Aspergillus* isolates resulted in fragments with band sizes of 598 bp and 650 bp, respectively. Sequenced amplicons queried on BLASTn tool in NCBI confirmed the identity of the putative isolates. Confirmed identifications revealed that the most abundant species were from section *Nigri* and included *A*. *niger*, *A*. *tubingensis*, *A*. *costaricaensis*, and *A*. *luchuensis* (Table 2). Species from section *Flavi* included *A*. *flavus*, *A*. *novoparasiticus*, *A*. *aculeatus*, and from section *Terrei*, species *A*. *terreus* was identified. Six of the isolates shared sequence identity with unidentified *Aspergillus* species (accessioned as “*Aspergillus* sp.”) (Table 2). The calmodulin gene was used to authenticate the 21 isolates from section *Flavi* identified through ITS and 28S rDNA. The results confirmed that all the isolates were from section *Flavi* (Figs 4 and 5). The calmodulin gene identified two sections; *Flavi* and *Nigri* with only two species: *A*. *flavus* and *A*. *aculeatus*, respectively, in contrast to the ITS and 28S rDNA results which revealed the presence of the two as well as *A*. *novoparasiticus* (Table 2). Klf07, Klf14, Klf16, Klf22, Lmu35, Lmu38, Kwl45 and Kwl50 which were identified as *A*. *flavus*, *A*. *aculeatus*, *A*. *aculeatus*, *A*. *novoparasiticus*, *Aspergillus sp*., *A*. *flavus*, *A*. *flavus* and *Aspergillus sp*. using ITS and 28S rDNA primers were classified as *A*. *aculeatus*, *A*. *flavus*, *A*. *flavus*, *A*. *flavus*, *A*. *flavus*, *A*. *aculeatus*, *A*. *flavus*, and *A*. *flavus* respectively via the calmodulin gene specific primers (Fig 5). Amplicons of the three genes of the aflatoxin biosynthesis pathway (*aflM*, *aflR* and *aflD*) resulted in band sizes of 536 bp, 500 bp and 400 bp, respectively (Supporting information 9, 10 in S1 File). The examination of the 63 *Aspergillus* isolates for the presence of any of three genes revealed that 5% (n = 3) contained *aflR* gene, 7% (n = 4) contained *aflD* gene, and 15.87% (n = 10) contained *aflM* gene (Supporting information 11 in S1 File). Twenty two percent (n = 14) of the isolates had at all the three the investigated genes, classifying the isolates as potentially aflatoxigenic. All the genes that amplified were confirmed to be from the section *Flavi* using ITS and 28S rDNA gene amplifications. Lamu County recorded the lowest number of *Aspergillus* isolates (3) that had at least one of the cluster genes while the highest number (8) was found in Kilifi County (Fig 6).

**Table 2:** Molecular confirmation of *Aspergillus* isolates, based on BLAST query of the ITS and 28s rDNA sequences, along with their GenBank accession numbers.

Isolate ID^a^	ITS Accession no.	Species ID^b^	Percent identity	Affiliated Accession No.	28S rDNA Accession No.	Species ID^b^	Percent Identity	Affiliated Accession No.
Klf02	OP737645	*A*. *niger*	99	ON241768.1	OP735641	*A*. *tubingensis*	99	MH870239.1
Klf03	OP737646	*A*. *flavus*	99	KX067886.1	OP735642	*A*. *flavus*	100	MT509808.1
Klf04	OP737647	*A*. *niger*	89	KF751668.1	OP735643	*A*. *tubingensis*	99	MH870293.1
Klf05	OP737648	*A*. *niger*	96	MF078659.1	OP735644	*A*. *niger*	99	FJ184995.1
Klf06	OP737649	*A*. *tubingensis*	99	MH045586.1	OP735645	*A*. *costaricensis*	100	NG069877.1
Klf07	OP737650	*A*. *niger*	97	MK108384.1	OP735647	*A*. *tubingensis*	99	MH866130.1
Klf08	OP737651	*A*. *niger*	97	OK353832.1	OP735648	*A*. *tubingensis*	99	MH870293.1
Klf09	OP737652	*A*. *aculeatus*	97	KP278205.1	OP735649	*A*. *niger*	99	MH872941.1
Klf12	OP737653	*A*. *flavus*	100	MN533863.1	OP735646	*A*. *novoparasiticus*	100	MT252035.1
Klf14	OP737639	*A*. *aculeatus*	99	KP965728.1	OP735631	*A*. *aculeatus*	99	MH870630.1
Klf17	OP737640	*A*. *niger*	95	MF078659.1	OP735634	*A*. *tubingensis*	99	MH870293.1
Klf18	OP737641	*A*. *tubingensis*	97	MG551279.1	OP735635	*A*. *tubingensis*	99	MH866130.1
Klf19	OP737642	*A*. *niger*	98	MH091026.1	OP735636	*A*. *sp*.	99	MN515385.1
Klf20	OP737643	*A*. *niger*	98	MF078659.1	OP735637	*A*. *costaricensis*	99	NG069877.1
Klf21	OP737644	*A*. *niger*	98	ON241768.1	OP735638	*A*. *niger*	99	GQ169752.1
Klf23	OP737632	*A*. *niger*	99	MH091026.1	OP735619	*A*. *costaricensis*	100	NG069877.1
Lmu26	OP737633	*A*. *tubingensis*	99	MG551279.1	OP735622	*A*. *luchuensis*	99	MH870355.1
Lmu27	OP737634	*A*. *niger*	96	MF078659.1	OP735623	*A*. *tubingensis*	99	MH870293.1
Lmu28	OP737635	*A*. *niger*	99	OK605290.1	OP735624	*A*. *tubingensis*	99	MH868003.1
Lmu30	OP737637	*A*. *flavus*	91	MT071404.1	OP735625	*A*. *oryzae*	99	KX958066.1
Lmu31	OP737638	*A*. *tubingensis*	99	MG551279.1	OP735626	*A*. *niger*	100	DQ914661.1
Lmu32	OP737627	*A*. *niger*	98	MF078659.1	OP735627	*A*. *costaricensis*	99	NG069877.1
Lmu33	OP737628	*A*. *niger*	98	KJ881377.1	OP735628	*A*. *niger*	100	MN153032.1
Lmu34	OP737629	*A*. *niger*	97	KJ881377.1	OP735629	*A*. *tubingensis*	100	MH870293.1
Lmu35	OP737630	*A*. *niger*	98	MK372989.1	OP735615	*Aspergillus sp*.	99	MN515285.1
Lmu36	OP737631	*A*. *flavus*	99	OK086056.1	OP735616	*A*. *flavus*	98	MT509808.1
Kwl39	OP737623	*A*. *flavus*	95	MK992255.1	OP735607	*A*. *flavus*	99	MT252035.1
Kwl40	OP737624	*A*. *niger*	99	MK307680.1	OP735608	*A*. *costaricensis*	100	NG069877.1
Kwl41	OP737625	*A*. *niger*	97	MF078659.1	OP735609	*A*. *tubingensis*	100	MH866130.1
Kwl42	OP737626	*A*. *niger*	98	MK886749.1	OP735610	*A*. *tubingensis*	99	MH870293.1
Kwl44	OP737613	*A*. *niger*	98	MH341159.1	OP735612	*A*. *costaricensis*	99	NG069877.1
Kwl46	OP737614	*A*. *niger*	93	KY702576.1	OP735614	*A*. *niger*	99	FJ184995.1
Kwl47	OP737615	*Aspergillus sp*.	99	KR183882.1	OP735596	*A*. *tubingensis*	99	MH870393.1
Kwl48	OP737616	*Aspergillus sp*.	92	OK210353.1	OP735597	*A*. *niger*	99	MW077307.1
Kwl49	OP737617	*A*. *flavus*	94	MK992254.1	OP735598	*A*. *novoparasiticus*	99	NG069972.1
Kwl51	OP737619	*A*. *niger*	98	KM222496.1	OP735599	*A*. *niger*	100	MK372923.1
Kwl52	OP737620	*A*. *niger*	97	ON241768.1	OP735600	*A*. *niger*	99	MH868943.1
Kwl53	OP737621	*A*. *niger*	96	MF152928.1	OP735601	*A*. *niger*	99	MH867698.1
Kwl54	OP737622	*Aspergillus sp*.	94	OK210353.1	OP735602	*A*. *tubingensis*	99	MH866130.1
Kwl55	OP737606	*A*. *niger*	98	MF078659.1	OP735603	*A*. *niger*	99	MH867830.1
Kwl57	OP737607	*A*. *niger*	99	MK372989.1	OP735605	*A*. *tubingensis*	100	MH870293.1
Kwl58	OP737608	*A*. *tubingensis*	96	KC020122.1	OP735606	*A*. *niger*	99	MW077307.1
Kwl59	OP737609	*A*. *niger*	98	MH091026.1	OP735591	*A*. *tubingensis*	100	MH870293.1
Kwl60	OP737610	*A*. *niger*	98	MF078659.1	OP735592	*A*. *tubingensis*	99	KY670607.1
Kwl61	OP737611	*A*. *terreus*	98	KJ685810.1	OP735593	*A*. *terreus*	99	MH877949.1
Kwl62	OP737612	*A*. *niger*	99	KJ881376.1	OP735594	*A*. *niger*	99	MW077307.1

^a^ Identifier assigned to each isolate based on sampling location.

^b^ Genus and/or species name obtained from BLASTn query through NCBI.

### Screening of aflatoxin presence in *Aspergillus* species isolates

The 25 of the 63 isolates that had the at least one of the three aflatoxin pathway genes were screened for aflatoxin production in coconut milk agar. In the UV at 365nm, the positive isolates fluoresced either green or blue (Supporting information 12 in S1 File). In the Ammonia test the AFs producing isolates changed colors from their original green mycelial pigmentation to yellow or dark yellow, pink, or reddish-brown (Supporting information 12 in S1 File). Of all the potential aflatoxin-producing species identified by amplification of the AFs pathway genes only 14 showed the presence of aflatoxins. The 14 isolates also showed presence of total aflatoxins in the ELISA assay (Supporting information 11, 13 in S1 File). The total aflatoxin absorbances (B/B0) ranged from 101.355 as the highest and the lowest being 71.023 (Supporting information 13 in S1 File).

### Diversity estimation of the *Aspergillus* isolates

Because one sequence had low quality, 62 28S rDNA and 63 ITS sequences were used to infer separate phylogenetic trees and determine the evolutionary relationships of the isolates. Sequences identified in section *Flavi* with >98% sequence similarity clustered together as one clade while the sequences of the section *Nigri* and having >98% sequence identity formed two clades (Fig 4). AMOVA results of the genetic variance across the Kilifi, Kwale and Lamu populations demonstrated that there were no significant differences in the genetic makeup both the 28S rDNA region (Table 3) and the ITS region (Table 4) among and within the three populations. The percentage of variation among the three populations was 0.71% and 0.85%, while percentage of variation within the populations was 99.29% and 99.15%, for the 28S rDNA and ITS regions, respectively. The fixation indices (F_ST_) for each respective region were 0.00715 and 0.00852, showing that that there was little genetic differentiation between the populations.

**Table 3:** The genetic variance of the *Aspergillus* isolates using the 28S rDNA.

Source of variation	d.f.	Sum of squares	Variance components	Percentage of variation (%)
Among populations	2	39.833	0.12594	0.71
Within populations	57	997.417	17.49854	99.29
Total	59	1037.250	17.62448	
Fixation index F_ST_ 0.00715				

Analysis of variance in the three populations and within the 60 populations of the 28S rDNA sequences. Comparisons were done using the AMOVA analysis in Arlequin. 28S rDNA AMOVA analysis (p<0.05) and significance test by 1023 permutations. Va and FST: p (rand. value > obs. value) = 0.24829, p (rand. value = obs. value) = 0.00098 and p = 0.24927±0.01438.

**Table 4:** The genetic variance of the *Aspergillus* isolates using the ITS region.

Source of variation	d.f.	Sum of squares	Variance components	Percentage of variation
Among populations	2	92.805	0.35116	0.85
Within populations	46	1880.665	40.88402	99.15
Total	48	1037.250	41.23518	
Fixation Index F_ST_: 0.00852				

Analysis of variance in the three populations and within the 49 populations of the ITS region sequences after alignment. Comparisons were done using the AMOVA analysis in Arlequin. ITS AMOVA analysis (p<0.05) and significance test by 1023 permutations. Va and FST: p (rand. value > obs. value) = 0.24927, p (rand. value = obs. value) = 0.00000 and p = 0.24927± 0.00000.

## Discussion

Moisture content is one of the most crucial quality factors for raw cashew nuts. In this study, the *Aspergillus* isolates were recovered from raw cashew nut kernels to evaluate their fungal contamination levels. [46] reported that temperature and moisture content are important factors that affect fungal growth rate and diversity in foodstuffs. Cashew nuts were first evaluated for moisture content and was correlated with growth of *Aspergillus* fungi. The results from this established that the moisture content of our cashew samples was below 10% which is safe for the storage of cashew nuts to avoid fungi growth [11]. The low range of moisture content also may infer a good whole kernel quality, which decreases as the moisture content of the cashew nuts increases [47]. The moisture content and the growth relationship of the isolated *Aspergillus* species from the sampled cashew nuts had a positive correlation (R^2^ = 0.21), indicating that the fungal growth accelerated as the moisture content of the cashew nuts increased (Fig 4). Similar finding showing a positive correlation of moisture content and fungal growth rate has been reported in cashew nuts [6] and in rice [27].

The average moisture content of the cashew nuts in the three counties was significantly different. The moisture content of the cashew nuts from Kilifi and Lamu counties were lower than that of the cashew nuts from Kwale County. The differences observed could result from several variables prior to sampling such as the moisture content of the soil on which the nut falls, or the climatic conditions of the field and the length of time the nut remains on the soil before collection [48]. Locations experiencing very high temperatures have lower moisture content and those stored in open areas for long have higher moisture content than those stored in locked and air-proof bags [48]. High moisture content in cashew nuts provides a good environment for fungal growth which could also result in the production and release of their secondary metabolites [11]. The study recommends limiting the comparison of the moisture content to nuts of the same variety, such as large or small, and not a combination of varieties, which may have different moisture content levels because the size of the cashew nuts may influence their kernel moisture content levels.

It is a difficult exercise to accurately identify and differentiate some *Aspergillus* species from each other based solely on morphology [49, 50]. Morphologically, five species were identified *A*. *niger*, *A*. *tubingensis*, *A*. *flavus*, *A*. *parasiticus* and *A*. *oryzae*. In the study, isolates representing five species were identified using the ITS region, and four isolates could not be identified to species using this region. Surprisingly, the 28S rDNA region expanded species representatives to nine with only two isolates remaining unidentified to species level. In both regions the sections identified were the same but in 28S rDNA identified some isolates identified as *A*. *niger* using ITS region into other species like *A*. *luchuensis*, and *A*. *costaricensis*.

Section *Nigri* species dominated the study cashew samples with *A*. *niger* being the predominant fungal contaminant. The *Aspergillus* species that were recovered in the cashew nuts includes *A*. *niger*, *A*. *tubingensis*, *A*. *aculeatus*, *A*. *luchuensis*, *A*. *costaricensis*, *A*. *flavus*, *A*. *parasiticus*, and *A*. *terreus*. The strains belong to three sections, the *Aspergillus* section *Flavi*, *Aspergillus* section *Nigri* and *Aspergillus* section *Terrei*. Identification of A. aculeatus using calmodulin gene specific primers may have been due misidentification using primers as previously been reported by [51] and/or as a result of the presence of the aflatoxin producing gene clusters in *Aspergilli* section *Nigri*, which are non-functional [52]. The section *Nigri* species were the most abundant followed by the section *Flavi*, and *A*. *niger* and its strains were more predominant. The results are in consonance with the findings of [46] on cashew nuts in Benin which also suggested that species from *Aspergillus* section *Nigri* (black *Aspergilli*) are among the most prevalent fungi that cause food spoilage and bio-deterioration of the nutrients in cashews and other nuts. Their potential to produce mycotoxins such as ochratoxins offers an additional risk to food safety [46]. However, the contributions of black *Aspergilli* to the fermentation industry, from production of organic acids and hydrolytic enzymes, make them of biotechnological significance [53]. For instance, *A*. *luchuensis* is widely utilized in Japan and China to produce koji, which is used in beverage fermentation [53]. Despite the capacity of *A*. *niger* to synthesize mycotoxins, it has made important contributions to industry and the U.S. Food and Drug Administration has classed products derived from this species as generally recognized as safe (GRAS) [8].

In the section *Flavi* species identified, *A*. *flavus* was most abundant. The results supports previous findings in cashew nuts in Nigeria by [54], groundnuts in Uganda [55], and in rice in Kenya [27]. Species of the *Aspergillus* section *Flavi* recovered from our cashew nuts samples were mostly *A*. *flavus* and *A*. *parasiticus*, which are the major aflatoxigenic species in cashew nuts [6, 46]. Another aflatoxigenic species, *A*. *nomius*, was not isolated from cashew nuts in the current study although it has been recovered from cashew nuts in South Africa [54]. In the section *Terrei*, *A*. *terreus* was identified. The results corroborate the findings reported in cashew nuts in Nigeria by [54]. *A*. *terreus* is well known for producing lovastatin, a cholesterol-lowering agent and other secondary metabolites such as territram, butyrolactones, and acetylaranotin with interesting bioactivities [56].

The gene cluster involved in aflatoxin production, which consists of the genes *AflR*, *AflS*, *AflP*, *AflD*, *AflM*, and *AflO*, regulates the capacity of fungal species to generate aflatoxins [56]. Any alteration in the sequences of the gene clusters involved in aflatoxin biosynthesis or the regulatory genes through insertions and deletions may cause *Aspergillus* isolates prone to aflatoxin production to become non-aflatoxigenic [57]. The isolates that showed amplification of the three selected genes were recorded as potentially aflatoxigenic. The use of the *AflD*, *AflM* and *AflR* genes was based on the key role they play in the biosynthesis of aflatoxins. The isolates that were not amplified by the selected genes were classified non-aflatoxigenic [58]. The non-aflatoxigenic nature could be due to the absence of the genes or major mutations within the regions flanking the genes or the genes regions that inhibit the aflatoxin production [59].

After screening for the presence aflatoxins in the potentially aflatoxigenic isolates, only isolates of the *Aspergillus* section *Flavi* were identified as aflatoxigenic. The species that were aflatoxigenic were *A*. *flavus* and *A*. *novoparasiticus* (n = 13) and one was identified as *Aspergillus sp*. From our study, the presence of the aflatoxin pathway genes was not consistent with the aflatoxin production of the isolates. The most predominant aflatoxin producers were *A*. *flavus*. The results agree with previous findings that *A*. *flavus* was the most abundant aflatoxigenic species in rice [27], maize [60] and peanuts [30, 58]. The study reports that the recovery of the aflatoxin-producing *Aspergillus* species can be associated with their ubiquitous nature and the potential of generating large numbers of spores that remain viable even in harsh environments [21]. In the current study, the contamination was attributed to the exposure of raw cashew nuts to fungal colonization during the harvesting period or post-harvest. Due to the toxic, teratogenic, and carcinogenic aflatoxins, there is need for assessment, detection, and quantification of the mycotoxin at each stage of cashew nuts processing.

*Aspergillus* species genetic diversity analysis in the cashew nuts is vital because it aids in the understanding of their local distributions. The diversity of both the ITS region and the 28S rDNA nucleotide sequences of the *Aspergillus* isolates recovered from our samples was estimated as they are known among the major mycotoxin producers in cashew nuts [32]. The phylogenetic analyses of both regions showed a similar distribution of the isolates into three clades. All the species of *Aspergillus* section *Flavi* clustered together while *Aspergillus* section *Nigri* species formed two clades regardless of the locality of the isolates. Results of the AMOVA demonstrated that the genetic variation within the populations was responsible for the genetic differences as compared to the variation among the populations both using 28S rDNA and ITS. The high genetic diversity in the *Aspergillus* isolates within the populations could be due to the farming practices in the different fields where the cashew nuts were collected from. Similar findings were also observed in *Aspergillus* species on groundnuts in Uganda by [55]. The study results also suggest a low genetic diversity among the three populations of *Aspergillus* isolates. This could have resulted from gene flow among the isolates in the different counties by the human activities causing the *Aspergillus* isolates contamination of the cashew nuts post-harvest. Secondly, the low genetic diversity could be due to the adaptation of the *Aspergillus* isolates to survive in adverse environments by the means of differential competition. The adaptation will make them to thrive together in similar climatic regions. Moreover, it could be an indication that most of the isolates shared similar insertions and deletions in both the ITS region and 28S rDNA sequences.

## Conclusions

Overall, results from the current study showed that raw cashew nuts collected from coastal Kenya had low and acceptable moisture content. However, fungi of the *Aspergillus* sections *Nigri* and *Flavi* were isolated as contaminants. The presence of isolates from section *Flav*i signified that there was contamination of the cashew kernels by aflatoxins. Additionally, the presence of section *Nigri* isolates could indicate the potential presence of other mycotoxins such as ochratoxins. Specific genes from the aflatoxin pathway found in all section *Flavi* isolates and the production of aflatoxins from then showed the cashew nuts could probably be contaminated with aflatoxins that render the products unsafe for food and feed. This call of the need to quantify the level of aflatoxins contamination in the cashew nuts. The genetic diversity of *A*. *flavus* and other fungi in various regions provides a valuable gene pool for potential for application in biocontrol to reduce the prevalence of aflatoxigenic fungi in the region through competitive exclusion mechanisms. Additionally, the identified aflatoxigenic *A*. *flavus* strains could be exploited to investigate the resistance of *Aspergillu*s species colonization and resultant aflatoxins and other mycotoxins production in different crop varieties grown in the region during breeding programmes. Further studies could investigate how and the conditions under which these cashew nut products are processed and whether due diligence is taken when preparing the raw materials for processing. This study calls for interventions including awareness creation, timely harvesting, rapid nut drying, appropriate storage facilities, sorting, and processing, insect control at pre-and post-harvest stages to reduce the incidences of aflatoxins contamination.

## Supporting information

S1 File (RAR)

S2 File Uncropped, and unadjusted images. (RAR)
